# Evaluating the toxicity and efficacy of the endophytic bacterium *Kosakonia* sp. ZO-Rh4 on antidiabetes and associated complications in a mouse model

**DOI:** 10.1016/j.bbrep.2025.102319

**Published:** 2025-10-19

**Authors:** Trang Thi Xuan Dai, Tran Chi Linh, Ta Lam Tai

**Affiliations:** a College of Natural Sciences, Can Tho University. Campus II, 3-2 Street, Ninh Kieu Ward, Can Tho City, 94000, Viet Nam; b Faculty of Medicine, Nam Can Tho University, 168, Nguyen Van Cu Ext Street, An Binh Ward, Can Tho City, 94000, Viet Nam; c Institute of Food and Biotechnology, Can Tho University, Campus II, 3-2 Street, Ninh Kieu Ward, Can Tho City, 94000, Viet Nam

**Keywords:** Acute toxicity, Alloxan, Anti-Diabetes, *Kosakonia* sp. ZO-Rh4, Sub-chronic toxicity

## Abstract

The endophytic bacterium *Kosakonia* sp. ZO-Rh4, isolated from ginger, exhibited antioxidant properties *in vitro*. However, its safety and efficacy have never been assessed *in vivo*. This study examined the toxicity of the ethyl acetate extract from *Kosakonia* sp. ZO-Rh4 (KE) in mice and its potential to treat diabetes and its complications. Acute toxicity was investigated with a single KE dosage of 5000 mg/kg body weight (b.w.). Sub-chronic toxicity was studied with a daily dosage of 400 mg/kg b.w. for 45 and 90 days. After 14 days of acute toxicity and 45 or 90 days of daily dosing at 400 mg/kg body weight, all parameters were found to be within normal limits. *In vitro* antidiabetic tests indicated that KE has a strong inhibitory effect on the α-amylase and α-glucosidase enzymes. An *in vivo* study was conducted on diabetic mice induced with alloxan monohydrate, measuring the mice's body weight and blood glucose levels weekly. Following the experiment, mouse blood was collected to measure lipid parameters and liver function markers. The liver, kidneys, and pancreatic tissues were evaluated for oxidative stress by measuring malondialdehyde and glutathione levels. KE lowered blood glucose and enhanced body weight depending on the dosage. Furthermore, KE possesses *anti*-dyslipidemic properties, can restore liver function, and mitigates oxidative stress in all examined organs by decreasing malondialdehyde and increasing glutathione. The study confirmed the relative safety of KE and its effectiveness in treating diabetes and its complications, potentially representing a new option for functional foods and pharmaceuticals.

## Highlights

• *Kosakonia* sp. ZO-Rh4 extract (KE) was obtained from the endophytic *Kosakonia* sp. ZO-Rh4.
• KE was found to be non-toxic in both acute and sub-chronic studies in mice.
• KE inhibited α-amylase and α-glucosidase, converting carbohydrates into glucose.
• KE had hypoglycemic, antidyslipidemic, and cardiovascular risk-lowering properties.
• KE reduced liver damage and oxidative stress in the liver, kidneys, and pancreas.

## Graphical abstract

**Figure:**
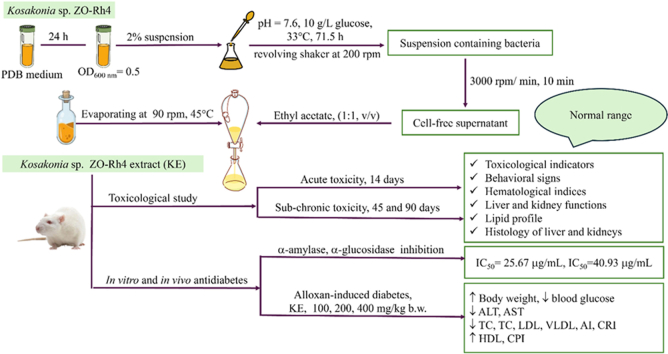

## 1 Introduction

Chronic high levels of blood glucose are the most characteristic of diabetes. Chronic hyperglycemia damages cells and tissues, leading to various complications associated with diabetes. Type 2 diabetes is the most common form, initially characterized by insulin resistance with compensatory hyperinsulinemia, followed by progressive β-cell dysfunction that leads to insufficient insulin secretion over time [1]. The global prevalence of type 2 diabetes has risen dramatically over the past three decades. According to the International Diabetes Federation (IDF), 463 million individuals had diabetes in 2019, and this number is projected to increase to 578 million by 2030 and 700 million by 2045 [2]. Modern treatments, such as insulin and oral hypoglycemic agents, are effective in controlling blood glucose but may cause adverse effects and have certain limitations [3].

Bacterial endophytes, which colonize plant tissues without causing visible disease symptoms, have received a lot of interest due to the potential for generating a variety of beneficial secondary compounds [4]. Secondary metabolites can block α-amylase and α-glucosidase, making them effective antidiabetic agents [5,6]. It has been demonstrated that endophytic bacteria, including *Streptomyces* [7,8], *Saccharomonas oceani* VJDS-3 from India's mangrove forest [9], *Amycolatopsis thermoflava* SFMA-103 [10], *Bacillus* sp. [11], and *Vibrio* sp. [12], inhibit α-amylase and α-glucosidase activity. Many compounds produced from *Actinoplanes utahensis* [13] have been shown to block α-amylase and α-glucosidase enzymes, including acarbose, which is used to treat diabetes. *Amycolatopsis thermoflava* produces 1-*O*-methyl chrysophanol, which inhibits carbohydrate-metabolizing enzymes *in vitro* and blood sugar levels *in vivo* [10]. α-glucosidase and α-amylase were significantly inhibited by the ethyl methoxycinnamate (ethyl (E)-3-4-methoxyphenyl) acrylate, isolated from *Saccharomonas oceani* VJDS-3 [9].

The genus *Kosakonia* (family Enterobacteriaceae) includes Gram-negative, rod-shaped, facultatively anaerobic bacteria often associated with plants as endophytes or rhizobacteria, where they contribute to nitrogen fixation, phosphate solubilization, and production of bioactive metabolites. From the rhizome of *Zingiber officinale* (ginger) in Vietnam, we isolated *Kosakonia* sp. ZO-Rh4, which shares 99.08 % *16S rRNA* sequence similarity with *K. arachidis* (GenBank accession no. PQ327934) and is characterized by yellow colonies, Gram-negative rod morphology, and strong antioxidant capacity with abundant polyphenol and flavonoid production. Our previous study demonstrated its *in vitro* antioxidant [14]. However, α-amylase and α-glucosidase inhibition *in vitro*, toxicological safety, and *in vivo* efficacy had not yet been investigated. Therefore, the present study aimed to evaluate the acute and sub-chronic toxicity, as well as the hypoglycemic, organ-protective, and lipid-modulating effects of *Kosakonia* sp. ZO-Rh4 extract in an alloxan-induced diabetic mouse model, thereby clarifying its therapeutic relevance in metabolic disorder management.

## 2 Materials and methods

### 2.1 Materials

The endophytic bacteria *Kosakonia* sp. ZO-Rh4 (Genbank accession no PQ327934.1) was identified earlier by 16S ribosomal RNA gene sequencing from the rhizome of *Zingiber officinale* [14] were pure cultured and stored in the Department of Biology, College of Natural Sciences, Can Tho University, Vietnam.

### 2.2 Methods

#### 2.2.1 Metabolite extraction

*Kosakonia* sp. ZO-Rh4 was cultured under ideal conditions in research by Dai et al. (2025) [14]. To extract secondary metabolites from *Kosakonia* sp. ZO-Rh4, 2 % of suspension containing bacteria with an optical density at 600 nm of 0.5, was cultured in potato dextrose broth (PDB) medium supplemented with 14.45 g/L d-glucose at 33 °C, pH of 7.58 for 71.5 h on a revolving shaker at 200 rpm. The culture broth medium was centrifuged at 3000 rpm for 30 min to obtain the cell-free supernatant containing bioactive compounds and discard the pellet. The resulting supernatant was mixed with ethyl acetate solvent at a 1:1 ratio (v/v) and stored at 4 °C overnight. The liquid ethyl acetate layer was then separated using a funnel and evaporated with a rotary evaporator operating at 90 rpm and 45 °C. The resulting crude extract was weighed to determine the extraction yield, which was calculated as the ratio of extract weight to the initial culture volume. The extraction yield of *Kosakonia* sp. ZO-Rh4 under these conditions was 1.7 g/L (0.17 % w/v). The extract was stored at −20 °C until used in further experiments.

#### 2.2.2 Experimental animals

The animals for this study were *Mus musculus* (Swiss albino) male mice, raised and housed in the Nha Trang Pasteur Institute and transported to the laboratory at Can Tho University. The animal study followed the Guideline for the Care and Use of Laboratory Animals (NIH Publication No. 85-23, revised 1996). Before the experiments began, the mice underwent a 7-day acclimation period to adapt to their new environment. During this time, they were housed in cages under controlled conditions, including a 12-h light/dark cycle, a temperature range of 29–30 °C, and a humidity level of 55–60 %, ensuring a stable and suitable habitat. The mice were given a conventional diet and unlimited access to clean water. Ninety-six of the 6-12 week-old mice weighing 25–30 g were randomly assigned 6 mice per group for the experiments [15].

#### 2.2.3 Evaluating acute toxicity and sub-chronic toxicity

##### 2.2.3.1 Acute toxicity

The acute toxicity test was conducted by OECD guideline 423 for chemical testing [15]. Treatment with oral *Kosakonia* sp. ZO-Rh4 extract (KE) in doses of 5, 50, 300, 2000, and 5000 mg/kg body weight (b.w.) was administered to five groups of six mice each, with a control group receiving a single dose of plain water, only on the first day of the study [16]. Mice were given an 8-h fast before treatment.

The mice used for this investigation were maintained in regular environmental conditions. All of the experimental animals were given similar water and meals. After receiving a single dosage of KE, mice were watched for 3 h, then twice a day (7 a.m. and 5 p.m.) for the next 14 days for any signs of toxicity, with mortality recorded. Any alteration in external appearance (skin or eye changes), symptoms of toxicity, including mucus membrane changes, respiratory depression, salivation, diarrhea, and behavioral changes such as sleep, coma, or lethargy, were noted. The lethal dosages for half of the mice (LD_50_), body weight, and blood glucose levels were examined after 14 days of treatment with 5000 mg/kg b.w. Finally, mice were sacrificed to obtain organs for weight determination and microscopic examination. Mouse heart blood was taken for biochemical and hematological analysis. The body and organs, including the liver, kidneys, and spleen, were weighed to calculate the organ weight/body weight ratio (relative organ ratio) [16].

##### 2.2.3.2 Sub-chronic toxicity

To evaluate the adverse effect of KE on mice, the sub-chronic toxicity was assessed according to the method reported by Sing et al. [16]. A total of 36 *Mus musculus* (Swiss albino) mice (6–8 weeks old, 25–30 g) were randomly assigned into two experimental sets corresponding to 45-day and 90-day observation periods (18 animals each). Each set included two groups: (i) treatment group (n = 12) administered KE orally at 400 mg/kg/day, and (ii) control group (n = 6) administered distilled water. Randomization was applied to allocate animals to groups. Investigators performing biochemical and hematological analyses were blinded to treatment allocation. Inclusion criteria were healthy mice without visible signs of disease, and no exclusion criteria were applied once allocated.

At the end of each observation period (day 45 or day 90), animals were fasted overnight, anesthetized with diethyl ether, and euthanized by exsanguination. Blood samples were collected for hematological and biochemical analysis. Organs (liver, kidney, spleen) were collected and weighed to determine relative organ weights. Liver and kidney tissues were fixed in 10 % neutral-buffered formalin, embedded in paraffin, sectioned at 5 μm, and stained with hematoxylin and eosin (H&E) for histopathological evaluation under light microscopy.

#### 2.2.4 Evaluating antidiabetic activity of KE

##### 2.2.4.1 In vitro antidiabetic Enzymatic assays

The assay of α-amylase inhibition activity was followed from Rawal et al. [17] with minor adjustments. The assays involved mixing 50 μL of KE (6.125–100 μg/mL) with 50 μL phosphate buffer (pH 7) and 50 μL α-amylase (3 U). Following careful mixing, the reaction mixture was held at 37 °C for 5 min. Then, add 50 μL of a 2 mg/mL starch solution and incubate at 37 °C for 15 min. Next, 1 M HCl and iodine were added with 200 and 300 μL, respectively, to stop the reaction. Finally, the absorbance of the reaction was recorded at 660 nm. The proportion of α-amylase inhibition of KE was calculated using the following formulas:

$$\alpha -\text{amylase}\;\text{inhibition}\;\left(\%\right)=100-\left[\left(1-A_{s}/A_{c}\right)\times 100\%\right]$$

where A_c_ = negative control absorbance and A_s_ = sample absorbance.

The effect of KE on the inhibition of the α-glucosidase was investigated using the protocol of Anh et al. (2021) [18], with only minor adjustments. Firstly, mix 20 μL of α-glucosidase enzyme (1 U) with 100 μL of 100 mM phosphate buffer (pH 6.8) and 40 μL of KE at various concentrations (6.125–100 μg/mL) and incubate for 15 min at 37 °C. Then, 40 μL of 5 mM pNPG (*p*-nitrophenyl-β-d-glucopyranoside) was added, and incubated for 20 min. Finally, 0.1 M of Na_2_CO_3_ (100 μL) was added to stop the reaction, and absorbance was measured at 405 nm. The inhibitory effectiveness of enzyme activity was established as:

$$\alpha -\text{glucosidase}\;\text{inhibition}=\left(1-A_{s}/A_{c}\right)\times 100\%$$

where A_c_ = negative control absorbance and A_s_ = sample absorbance.

The negative and positive controls were phosphate buffer and acarbose, respectively, and were done in the same way as the extract in both enzyme assays.

##### 2.2.4.2 Evaluating the antidiabetic activity of KE on alloxan-induced diabetic mice

###### 2.2.4.2.1 Diabetic mice induction

After fasting for 18 h, mice were given alloxan monohydrate (AM) intraperitoneally with a daily dose of 135 mg/kg b.w. for three days. Blood glucose levels were recorded using the ACCU-CHEK® Active (ROCHE, Vietnam) after 7 days from the last injection. Mice having glucose levels equal to or more than 12 mmol/L were chosen as diabetic mice for the study [19].

There were six mouse groups (n = 6), including Group I, which consisted of normal mice (NM) consuming distilled water orally. Group II: diabetic mice (DM) received purified water orally. Group III: diabetic mice were treated with Metformin at a daily dose of 108 mg/kg b.w. (DM + Met). Diabetic mice in Groups IV, V, and VI were administered orally with 100 (DM + KE, 100), 200 (DM + KE, 200), and 400 (DM + KE, 400) mg KE/kg b.w daily. The study was performed in 4 weeks.

###### 2.2.4.2.2 Biochemical analysis and histopathological examination

After 28 days of testing, mice were starved for 24 h and sacrificed using anesthetic diethyl ether. Blood and organs were collected for further analysis and microscopic observation. Glucose levels were determined by ACCU–CHEK® Active. Plasma was obtained from the blood sample after centrifugation at 3000 rpm for 10 min. Total cholesterol (TC), triglyceride (TG), low-density lipoprotein cholesterol (LDL), high-density lipoprotein cholesterol (HDL), and liver function enzymes alanine transaminase (ALT), aspartate transaminase (AST) of plasma, were determined by using the semi-automated machine Erba Chem-7 (Erba, Germany). Very-low-density lipoprotein cholesterol (VLDL) was calculated by the formula [20]: VLDL = TG/2.2. Furthermore, cardioprotective index (CPI), atherogenic index (AI), and coronary risk index (CRI) were determined by the following formula [21]: CPI = HDL/LDL, AI = [TC - HD]/[HDL] and CRI = TC/HDL.

The effect of KE on the generation of lipid peroxidation and endogenous antioxidants in the liver, kidneys, and pancreas of mice was determined by the analysis of malondialdehyde (MDA) [22] and glutathione (GSH) [23], respectively. The concentration (nM/g tissue) of MDA and GSH was estimated using the linear regression equations for MDA and GSH standards.

#### 2.2.5 Ethical approval and animal welfare

This study will adhere to the ethical standards established by the US National Institutes of Health Guide for the Care and Use of Laboratory Animals (NIH Publication No. 85-23, revised 1996) and was approved by Can Tho University's Animal Ethics Committee (CTU-AEC) with code number CTU-AEC24001.

#### 2.2.6 Analysis of data

All *in vitro* biochemical determinations were carried out in triplicate, while animal experiments were performed once with appropriate group sizes as described. The experimental data were reported as means and standard deviations (SD) or standard error of the mean (SEM). Minitab 16 software was used to analyze the statistical results (ANOVA-Tukey's). Correlation analyses were carried out by using Pearson's correlation analysis. A p-value less than 0.05 was a significant difference. Graphs were plotted using GraphPad Prism (GraphPad Software, LLC, US).

## 3 Results

### 3.1 Evaluating KE's acute and sub-chronic toxicity

**Fig. 1:**
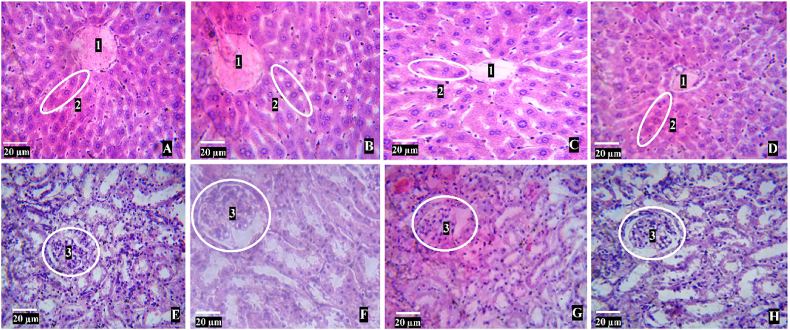
(A), (B), (C), and (D) Cross sections of the livers of control mice and mice treated with KE 5000 mg/kg for 14 days, 400 mg/kg for 45 days, and 90 days, respectively, revealing well-preserved central veins (1) and liver plates (2). (E), (F), (G), and (H) Cross sections of the kidneys of a control mouse and mice treated with KE 5000 mg/kg for 14 days, 400 mg/kg for 45 and 90 days, respectively, exhibiting preserved glomeruli (3) and tubules (hematoxylin-eosin).

All mice treated with KE at various doses, with the maximal dose of 5000 mg/kg b.w., exhibited no toxicological indications or behavioral changes (Table 1). All of the experimental mice appeared to have normal skin and eyes. Mice slept and urinated normally, showing no signs of lethargy, stupor, or drowsiness. KE-treated mice ate and drank similarly to normal control mice. The body weight, relative liver, kidneys, and spleen of KE-treated mice were not significantly different from normal mice. Furthermore, 14 days after orally administering 5000 mg/kg b.w. KE to mice, hematological and biochemical indices, and liver and kidney histology, revealed no significant adverse effects (Table 2, Fig. 1).

**Table 1:** General appearance and behavior for acute toxicity in KE treatment at a dose of 5000 mg/kg b.w. and control groups of mice after 14 days of the experiment.

Paradigm	Observation	Control	5000 mg/kg b.w
External appearance	Mortality	None	None
	Change in skin	No change	No change
	Change in eyes	No change	No change
Sign of toxicity	Change in the mucus membrane	No change	No change
	Respiratory depression	Not observed	Not observed
	Salivations	No effect	No effect
	Diarrhea	No effect	No effect
Behavioural change	Sleep	No effect	No effect
	Coma	Not observed	Not observed
	Lethargy	No effect	No effect
	Urination	No effect	No effect
	Drowsiness	Not present	Not present
Food and water intake	Normal	No effect	No effect

**Table 2:** Parameters for body weight, relative organ ratio, biochemical, and hematological markers of the acute and sub-chronic toxicity.

Parameters	Acute toxicity		Sub-chronic toxicity (45 days)		Sub-chronic toxicity (90 days)	
	Normal mice	KE, 5000	Normal mice	KE, 400	Normal mice	KE, 400
Body weight (g)	33.24 ± 2.79	32.70 ± 0.73	33.04 ± 0.34	32.98 ± 0.53	33.32 ± 0.18	33.30 ± 0.49
Relative ratio of liver (%)	6.66 ± 0.11	6.54 ± 0.25	3.93 ± 0.18	4.12 ± 0.34	4.19 ± 0.17	4.09 ± 0.38
Relative ratio of kidneys (%)	4.83 ± 0.53	4.79 ± 0.45	1.00 ± 0.05	1.00 ± 0.03	1.00 ± 0.03	0.98 ± 0.04
Relative ratio of spleen (%)	0.93 ± 0.11	0.92 ± 0.02	2.84 ± 0.05	2.83 ± 0.09	2.81 ± 0.04	2.83 ± 0.02
Blood glucose (nmol/L)	2.93 ± 0.22	2.90 ± 0.06	6.50 ± 0.16	6.42 ± 0.19	6.46 ± 0.23	6.42 ± 0.18
Urea (mmol/L)	3.89 ± 0.91	3.85 ± 0.54	3.43 ± 1.31	4.37 ± 0.69	6.53 ± 1.59	6.75 ± 1.08
Creatinine (μmol/L)	81.40 ± 22.19	95.20 ± 16.07	73.17 ± 15.98	82.67 ± 5.32	124.00 ± 33.81	132.83 ± 27.31
Cholesterol (mmol/L)	3.66 ± 0.39	3.62 ± 0.57	3.62 ± 0.56	3.68 ± 0.31	2.90 ± 0.99	2.03 ± 0.47
HDL (mmol/L)	0.87 ± 0.18	0.99 ± 0.15	0.87 ± 0.23	0.65 ± 0.24	0.65 ± 0.29	1.16 ± 0.73
Triglyceride (mmol/L)	1.22 ± 0.53	0.84 ± 0.23	1.33 ± 1.01	0.63 ± 0.38	2.54 ± 1.67	2.95 ± 1.44
AST (U/L)	82.40 ± 15.39	99.20 ± 24.72	68.33 ± 22.29	71.33 ± 11.08	118.50 ± 44.12	92.50 ± 41.91
ALT (U/L)	82.80 ± 13.03	106.60 ± 27.79	121.67 ± 20.41	135.00 ± 10.49	165.33 ± 34.52	114.00 ± 43.34
WBC	9.88 ± 2.15	11.76 ± 1.64	5.83 ± 0.60	4.90 ± 1.13	6.88 ± 1.49	7.40 ± 1.15
NEU (%)	5.62 ± 3.22	5.44 ± 1.49	3.73 ± 1.54	3.25 ± 0.83	3.63 ± 3.03	4,02 ± 1,71
LYM (%)	80.44 ± 8.30	81.12 ± 3.11	85.83 ± 4.05	84.73 ± 2.15	82.58 ± 6.67	86.60 ± 40.00
MONO (%)	13.94 ± 5.10	13.44 ± 1.78	10.43 ± 3.10	12.02 ± 1.55	13.40 ± 5.08	9.77 ± 0.99
RBC (10^6^/μL)	9.05 ± 0.82	8.16 ± 0.94	9.73 ± 0.31	9.74 ± 0.93	9.42 ± 0.64	8.37 ± 0.42∗
HRB (g/L)	146.00 ± 13.10	137.60 ± 17.24	151.83 ± 2.90	146.67 ± 12.80	140.33 ± 7.94	131.67 ± 4.84∗
HCT	48.74 ± 5.86	44.26 ± 6.52	52.93 ± 2.19	50.25 ± 6.45	46.67 ± 1.69	42.22 ± 1.66∗
MCV	53.80 ± 2.78	54.00 ± 2.45	54.50 ± 2.07	51.33 ± 2.34∗	49.50 ± 2.51	57.17 ± 15.61
MCH	16.14 ± 0.71	16.84 ± 0.75	15.62 ± 0.60	15.07 ± 0.5	14.95 ± 1.40	15.72 ± 0.96
MCHC	30.26 ± 1.02	31.16 ± 1.09	28.75 ± 1.28	29.45 ± 1.36	29.83 ± 1.73	31.13 ± 1.45
PLT	839.20 ± 137.90	845.80 ± 136.5	776.67 ± 115.62	530.67 ± 43.63∗	1139.70 ± 617.40	725.20 ± 184.90

Values are expressed as MEAN ± SEM (n = 6). ∗ Significant difference (P < 0.05) between normal and KE-treated group in the acute (or sub-chronic for 45 or 90 days) toxicity. HDL: high-density lipoprotein cholesterol, AST: aspartate serum transaminase, ALT: alanine transaminase, WBC: white blood cells, NEU: neutrophil, LYM: lymphocyte, MONO: monocyte, RBC: red blood cell, HGB: hemoglobin, HCT: Hematocrit, MCV: mean corpuscular volume, MCH: mean corpuscular hemoglobin, MCHC: mean corpuscular hemoglobin concentration, PLT: platelet.

During the 45 and 90-day study periods, 400 mg/kg/day of KE did not cause death. As expected, body weight and liver, kidney, and spleen relative ratios were similar to the normal. In a sub-chronic toxicity study, there were no changes in blood glucose, lipid profiles, renal and hepatic function (Table 2). The animal liver and kidney histology tests of the mice groups treated with KE in sub-chronic toxicity at 400 mg/kg for 45 and 90 days of KE demonstrated a wide range of tissue normalities (Fig. 1).

The blood hematological analysis found no significant hematological alterations because the majority of these indicators were within normal levels during the 45 and 90 days of the investigation. The 45-day trial resulted in substantial differences in mean corpuscular volume (MCV) and platelet count (PLT), while red blood cell count (RBC), hemoglobin (HGB), hematocrit (HCT), and mean corpuscular hemoglobin (MCH) revealed significant alterations compared to the normal 90-day chronic research (P < 0.05). There were no changes in animal health or weight loss during the sub-chronic study. These findings suggest that KE is not dangerous, but it does create a small change, with lower RBC, HGB, and HCT counts and higher MHC and PLT levels.

### 3.2 Effects of KE on antidiabetic activity *in vitro*

**Fig. 2:**
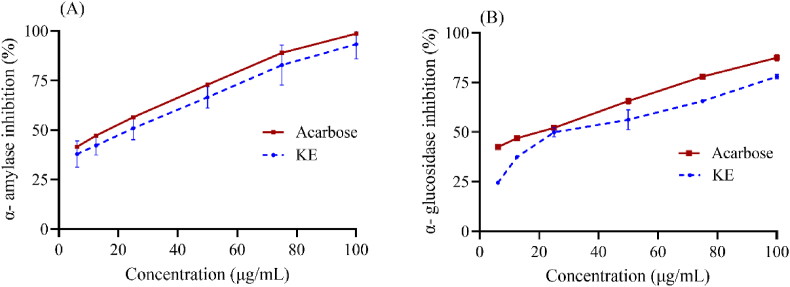
α-amylase (A) and α-glucosidase (B) inhibitory activities of KE.

The enzyme inhibition activity of α-amylase and α-glucosidase was used as a primary assay to evaluate antidiabetic efficacy. Inhibition of α-amylase and α-glucosidase capacity depended on the KE concentration (Fig. 2). KE had an IC_50_ (μg/mL) of 25.67 (y = 0.6185x+34.122, R^2^ = 0.9911) for α-amylase and 40.93 (y = 0.498x+29.617, R^2^ = 0.9254) for α-glucosidase. In comparison to acarbose, a positive reference, the IC_50_s were 16.25 (y = 0.6176x+39.963, R^2^ = 0.9898) and 19.90 (y = 0.4811x+40.427, R^2^ = 0.9965), respectively.

### 3.3 The effects of KE on hypoglycemia, anti-dyslipidemia, and antioxidant *in vivo* in alloxan-induced diabetic mice

#### 3.3.1 Effects of KE on the Mice's body weight and blood glucose levels

**Fig. 3:**
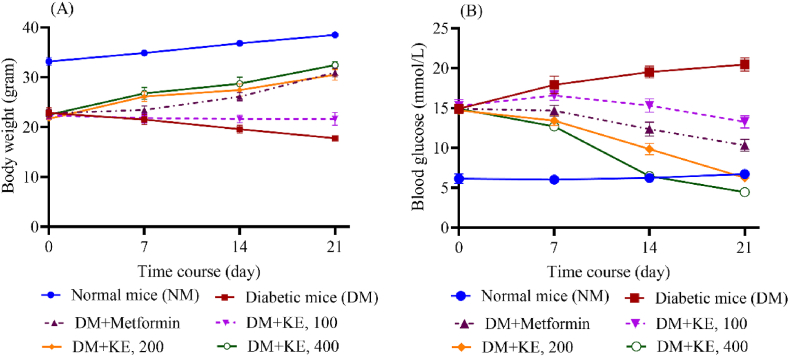
Effect of KE on mice's body weight (A) and blood glucose levels (B) in alloxan-induced diabetic mice. The data are presented as Mean ± SEM (n = 6), with P < 0.05 indicating significant differences between the groups treated with Metformin or KE and the untreated diabetic group at 7, 14, or 21 days, excluding diabetic mice treated with KE at a dose of 100 mg/kg.

Body weight and blood glucose levels are the most important factors in evaluating diabetic status in mice. Untreated diabetic mice had significantly lower body weights time-dependently, decreasing from 22.84 ± 1.03 g to 17.78 ± 0.33 g, compared to normal mice (33.16 ± 0.77 g). KE-treated diabetic mice with 100, 200, and 400 mg/kg b.w. dosages were restored to normal body weight status, 21.66 ± 1.29, 30.50 ± 1.05, 32.44 ± 0.68, respectively; at a dose of 400 mg/kg of mice's body weight was no significant difference from the normal. Compared to the positive control, Metformin, the effects of KE at 200 and 400 mg/kg b.w. dosages obtained the same results after 21 days of treatment. However, KE at a dose of 100 mg/kg did not significantly recover body weight (Fig. 3A).

Fig. 3B illustrates the effects of KE on the blood glucose levels of mice. In untreated diabetic mice, glycemia increased significantly during the experiment (from 14.92 ± 0.59 to 20.46 ± 0.83 mmol/L). Treatment with KE at doses of 200 and 400 mg/kg b.w. produced marked reductions in blood glucose, reaching 6.26 ± 0.11 and 4.46 ± 0.37 mmol/L, respectively, after 21 days, values close to those observed in normal mice (6.74 ± 0.06 mmol/L). At these higher doses, KE lowered glycemia more effectively than Metformin (108 mg/kg, 10.34 ± 0.74 mmol/L). However, administration of KE at 100 mg/kg did not result in significant glucose reduction compared to diabetic controls, indicating that the antihyperglycemic efficacy of KE is dose-dependent. These findings suggest that KE has promising antidiabetic potential, but further work is needed to compare its efficacy directly with Metformin at equivalent doses.

#### 3.3.2 Effects of KE on the lipid profile and cardiovascular index

**Fig. 4:**
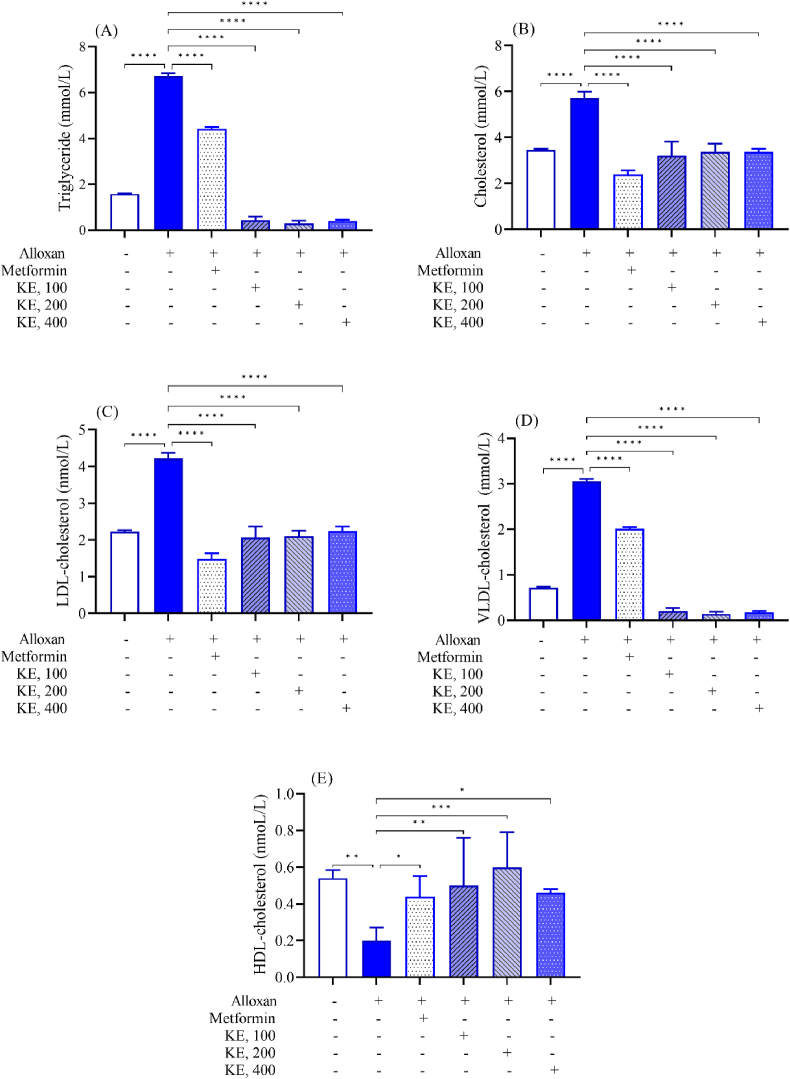
Effects of KE on lipid profile of alloxan-induced diabetic mice. (A): triglyceride, (B): cholesterol, (C): LDL-cholesterol, (D): VLDL-cholesterol, (E): HDL-cholesterol. Data are present as Mean ± SEM (n = 6); ∗P < 0.05, ∗∗P < 0.01, ∗∗∗P < 0.001, ∗∗∗∗P < 0.0001 compared to untreated diabetic mice.

**Fig. 5:**
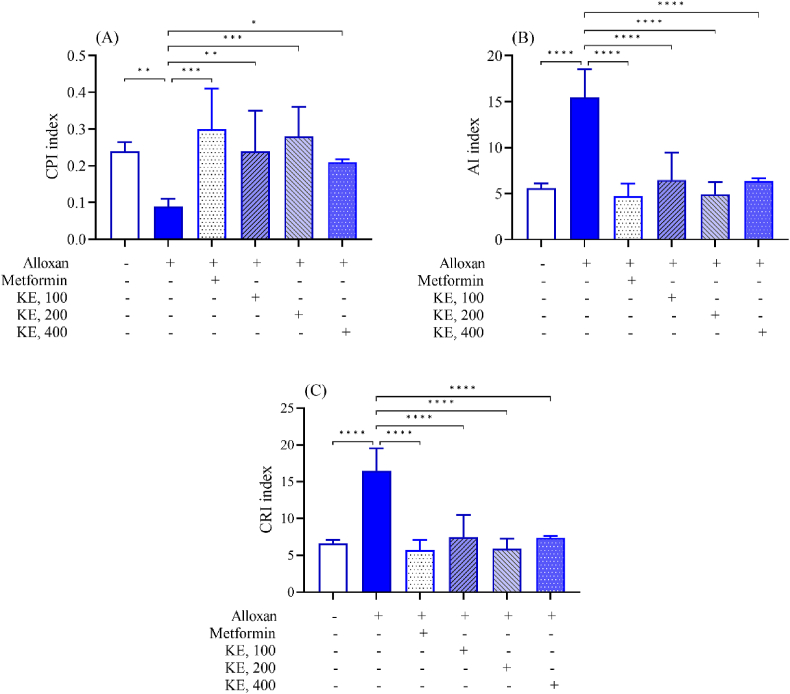
Effects of KE on cardioprotective (CPI), atherogenic (AI), and coronary risk (CR) indices in alloxan-induced diabetic mice. (A): CPI, (B): AI, (C): CR. Data are present as Mean ± SEM (n = 6); ∗P < 0.05, ∗∗P < 0.01, ∗∗∗P < 0.001, ∗∗∗∗P < 0.0001 compared to untreated diabetic mice.

Diabetic mice had significantly higher serum levels of total triglycerides (TG) (6.72 ± 0.13 mmol/L), total cholesterol (TC) (5.72 ± 0.28 mmol/L), low-density lipoprotein cholesterol (LDL) (4.22 ± 0.15 mmol/L), and very low-density lipoprotein cholesterol (VLDL) (3.05 ± 0.06 mmol/L) and lower levels of high-density lipoprotein cholesterol (HDL) (0.20 ± 0.07 mmol/L) than normal controls (1.58 ± 0.08, 3.46 ± 0.11, 2.23 ± 0.08, 0.72 ± 0.04, and 0.54 ± 0.11 mmol/L for TG, TC, LDL, VLDL, HDL, respectively) (Fig. 4), indicating that alloxan-induced diabetes causes dyslipidemia. Furthermore, in diabetic mice, cardioprotective was reduced 2.67-fold (CPI = 0.09 ± 0.02), while atherogenic (AI) (15.48 ± 3.06) and coronary risk (CR) (16.48 ± 3.06) increased 2.76- and 2.49-fold, respectively; suggesting that hyperglycemia and dyslipidemia increase the risk of cardiovascular disease (Fig. 5). In diabetic mice, KE demonstrated antidyslipidemic activity at dosages of 100, 200, and 400 mg/kg b.w., combined with lowering the risk of cardiovascular problems (Fig. 4, Fig. 5).

#### 3.3.3 Effects of Kosakonia sp. ZO-Rh4 extract on hepatic function

Mice treated with KE at all experiment doses (100, 200, and 400 mg/kg b.w.) had considerably lower enzymes of AST (78.0 ± 11.0, 72.0 ± 8.4, and 64.0 ± 8.9 U/L) and ALT (122.0 ± 22.8, 112.0 ± 11.0, and 104.0 ± 21.9 U/L) than the untreated diabetic group (AST: 4738.4 ± 643.9, ALT: 3648.2 ± 393.7 U/L) (P < 0.05; Table 3). Notably, mice treated with KE at 100 mg/kg doses had lower ALT levels than the Metformin and normal control groups. In contrast, AST concentrations of the Metformin group were similar to the normal control group (Table 3), indicating that KE at a dose of 100 mg/kg b.w. could improve AST and ALT within normal ranges.

**Table 3:** Effects of KE on indicators for organ damage in alloxan-induced diabetic mice.

Groups	Marker enzymes for liver damage		MDA (nM MDA/g tissue)			GSH (nM GSH/g tissue)		
	AST (U/L)	ALT (U/L)	Liver	Kidneys	Pancreas	Liver	Kidneys	Pancreas
Normal mice (NM)	273.8 ± 4.1^b^	136.2 ± 46.0^b^	2.47 ± 0.05^b^	12.38 ± 0.07^b^	2.68 ± 0.07^b^	535.1 ± 92.3^d^	227.6 ± 53.3^d^	412.1 ± 53.3^d^
Diabetic mice (DM)	3648.2 ± 393.7^a^	4738.4 ± 643.9^a^	15.95 ± 0.17^a^	80.52 ± 0.13^a^	15.86 ± 0.13^a^	104.6 ± 53.3^e^	73.8 ± 7.7^e^	106.4 ± 43.3^f^
DM + Met	243.0 ± 38.2^b^	121.0 ± 22.5^b^	2.13 ± 0.14^c^	11.67 ± 0.02^c^	1.97 ± 0.02^c^	1303.8 ± 266.3^c^	873.3 ± 53.3^c^	811.8 ± 159.8^cd^
DM + KE, 100	78.0 ± 11.0^c^	122.0 ± 22.8^b^	2.14 ± 0.05^c^	11.52 ± 0.05^c^	1.82 ± 0.05^c^	1672.8 ± 53.3^c^	904.1 ± 92.3^c^	1457.6 ± 92.3^c^
DM + KE, 200	72.0 ± 8.4^c^	112.0 ± 11.0^b^	1.54 ± 0.03^d^	4.50 ± 0.10^d^	1.27 ± 0.10^d^	3517.8 ± 140.9^b^	2595.3 ± 140.9^b^	2902.8 ± 416.9^b^
DM + KE, 400	64.0 ± 8.9^c^	104.0 ± 21.9^b^	0.52 ± 0.14^e^	2.05 ± 0.15^e^	0.62 ± 0.08^e^	7207.9 ± 140.9^a^	5362.9 ± 140.9^a^	6592.9 ± 436.2^a^

Values are expressed as MEAN ± SEM (n = 6). Different letters in the column show statistically significant (P < 0.5). NM: normal group; DM: untreated diabetic group; DM + Met: diabetic mice treated with Metformin at a dose of 108 mg/kg b.w; DM + KE, 100, DM + KE, 200, DM + KE, 400: diabetic mice treated with KE at doses of 100, 200, and 400 mg/kg. AST: aspartate serum transaminase, ALT: alanine serum transaminase, MDA: malondialdehyde, GSH: reduced glutathione.

#### 3.3.4 Effects of KE on the oxidative stress of diabetic mice

Table 3 displays the amounts of MDA (lipid peroxidation product) and GSH (reduced glutathione, an endogenous antioxidant) in the liver, kidneys, and pancreas. Untreated diabetic mice had significantly greater about 6-fold MDA levels in liver (15.95 ± 0.17), kidneys (80.52 ± 0.13), and pancreas (15.86 ± 0.13), and lower 5.1-, 3.1-, and 3.9-fold GSH levels in liver (104.6 ± 53.3), kidneys (73.8 ± 7.7), and pancreas (106.4 ± 43.3), respectively, than the control group across all organs (P < 0.05). These results indicated that diabetic mice experienced severe oxidative stress occurs various organs. Following a 21-day course of treatment with 100 mg/kg of KE or Metformin, nearly all of the metrics returned to normal (P < 0.05).

## 4 Discussion

Mortality is an important element in evaluating toxic effects since it exposes the direct result of severe poisoning [24]. All parameters were normal in all mice in the 5000 mg/kg b.w. treatment group during the study period, indicating that the KE extract had no major physiologic effects [25]. The LD_50_ for KE is higher than 5000 mg/kg, indicating that the KE extract is not harmful to mice. The Globally Harmonized System (GHS) of Classification and Labelling of Chemicals considers compounds with an LD_50_ value greater than 2000 mg/kg to be reasonably safe [26] LD_50_ values above 5000 mg/kg are deemed relatively safe for acute exposure under the GHS standard. Based on the LD_50,_ a dosage of 400 mg/kg is utilized for sub-chronic toxicity.

Sub-chronic toxicity studies of 90 days are appropriate for the extract [27]. The key indicators used to assess the first symptoms of poisoning in mice are general behavior, mortality, and body weight change [28]. The animal's body weight loss of more than 20 % is considered essential and has been designated as a humane endpoint in numerous international guidelines [28]. Determining weight growth or relative organ weight is critical for identifying potential harm caused by extract-related hazardous chemicals. The weight of the injured organ would vary based on the level of toxicity and its proportion to the body weight. In this sub-chronic toxicity investigation, the KE extract at a dose of 400 mg/kg caused no change in clinical signs, death, or morbidity. Additionally, the animals gained weight during the 45 and 90-day research.

To determine the toxicity of any extracts or chemicals, it is critical to analyze kidney and liver functioning, which are required for an organism's survival [28]. Renal function may be diagnosed using blood indicators like creatinine and urea. Elevated levels of these indicators in the bloodstream can result in impaired renal function and failure [28]. Creatinine and urea levels were found to be identical to those of the control group. Histopathological studies of the kidneys confirmed this finding, indicating normal anatomy (Fig. 1).

Liver damage was indicated by abnormally high levels of ALT and AST indicators [29]. These enzymes are produced predominantly by hepatocytes. Increased liver enzyme levels may indicate hepatocellular toxicity. However, unlike AST, ALT is a more sensitive sign of liver disease or injury. AST can also be found in other organs such as the kidneys, heart, testes, and skeletal muscles [30]. Our findings showed that the ALT and AST levels were within normal ranges, indicating that KE at a dosage of 400 mg/kg for 45 and 90 days of oral administration had no negative effects on liver function. Liver histopathological examination corroborated this discovery, showing normal anatomy (Fig. 1).

In both the 45 and 90-day periods of the research, the blood glucose and lipid profiles of mice treated with 400 mg/kg KE did not alter appreciably when compared to normal animals. Hematological indices revealed identical results between 400 mg/kg b.w. KE treated mice and normal mice, except MCV and PLT, for 45 days, and RBC, HBG, and HCT for 90 days (Table 2). Hematological indicators exhibited non-toxicological changes, with no significant relationships found between the data. The changes were considered spontaneous because the ratio fluctuations were typically less than 10 % and did not coincide with changes in liver or kidney biomarkers.

*In vitro* antioxidant activity was demonstrated by *Kosakonia* sp. ZO-Rh4, which was isolated from Zinger rhizomes [14]. According to this study, *Kosakonia* sp. ZO-Rh4 can be utilized as a natural source because the ethyl acetate extract of the cell-free supernatant exhibited neither acute nor sub-chronic toxicity. To learn more about the bioactivity of *Kosakonia* sp. ZO-Rh4, its antidiabetic efficacy was investigated both *in vitro* and *in vivo*. Alpha-amylase converts starch into oligosaccharides, and α-glucosidase enzyme catalyzes oligosaccharides into glucose. As a consequence, these two enzyme inhibition assays are widely used to screen for medicines with antidiabetic characteristics [31]. KE's potent α-amylase and α-glucosidase inhibitory activity (Fig. 2) indicates its antidiabetic potential by reducing blood glucose levels postprandial.

The most common symptoms of diabetes mellitus are high levels of blood glucose and decreased body weight due to insulin resistance or insufficient insulin synthesis [1]. KE supplementation significantly increased body weight and reduced blood glucose levels in diabetic mice compared to the untreated diabetic control mice. The *in vivo* investigation in this experiment is compatible with the *in vitro* study, which found that KE includes α-amylase and α-glucosidase inhibitors as well as antioxidants [14].

Diabetes mellitus is characterized by elevated TC, TG, LDL, and VLDL levels, and reduced HDL levels [32], which are caused by altered lipid and carbohydrate metabolism. This increases the cardiovascular risk, an atherogenic problem, and coronary heart disease, as demonstrated by a drop in CPI and a rise in AI and CRI [32]. These anomalies are linked to the emergence of cardiovascular disorders in diabetic mellitus as the condition worsens [33]. According to this study, KE-treated diabetic mice had less cardiac disease and significantly lower serum levels of TC, TG, LDL, and VLDL, as well as AI and CRI. They also showed greater levels of HDL and CPI. These data indicate that KE may affect decreasing or preventing lipid metabolism problems associated with diabetes.

Liver damage is also fairly prevalent in chronic hyperglycemia. Alloxan-induced diabetic mice show an increase in liver function enzymes such as ALT and AST [34]. KE's treatment of mice after 21 days significantly restored the levels of ALT and AST to normal control. Moreover, hyperglycemia is associated with increased oxidative stress and chronic inflammation, which contribute to severe hyperglycemia. Conversely, excessive glucose generates free radicals, which initiate long-term inflammation. The interplay of inflammation, oxidative stress, insulin resistance, and hyperglycemia perpetuates this vicious cycle, raising the risk of complications from diabetes [35]. Malondialdehyde (MDA), which is formed during the oxidation of lipids, while glutathione (GSH) is an endogenous antioxidant. The increase of MDA and the decrease of GSH reflect the oxidative stress in the organs. Untreated diabetic mice's liver, kidneys, and pancreas showed high MDA and low GSH, which is indicative of severe oxidative stress that damages organs and leads to complications. MDA levels were lowered after KE therapy at all doses, but GSH levels increased, which was beneficial. The biochemical findings indicate that KE treatment significantly restored liver function in diabetic mice, as evidenced by reduced ALT/AST activities, decreased MDA, and increased GSH. These results support the view that antioxidant activity represents a principal mechanism by which KE protects hepatic tissue. Nevertheless, additional pathways may also be involved. Improved lipid regulation by KE likely alleviates hepatic steatosis and reduces lipotoxicity, indirectly decreasing inflammatory signaling within the liver. Moreover, polyphenols and flavonoids, which are abundant in KE, have been widely reported to suppress NF-κB–mediated pro-inflammatory cytokine production and to inhibit TGF-β–driven activation of hepatic stellate cells, thereby exerting anti-fibrotic effects. Based on this evidence, we hypothesize that KE exerts hepatoprotection not only through antioxidant and metabolic effects, but also potentially through anti-inflammatory and anti-fibrotic mechanisms. However, because our present study did not measure inflammatory cytokines, fibrotic markers, or histological changes, further targeted studies are needed to validate these additional pathways [[36], [37], [38], [39]].

Although several members of the genus *Kosakonia* have been reported as plant-associated bacteria with growth-promoting activities, certain species have also been described as opportunistic human pathogens. This raises concerns regarding the safety of using *Kosakonia* strains in food or pharmaceutical applications. However, *Kosakonia* sp. ZO-Rh4 was isolated from the rhizome of *Zingiber officinale* and did not produce any acute or sub-chronic toxicity in mice at the tested doses. Moreover, no pathogenic symptoms were observed during animal experiments. Nevertheless, further genomic and toxicological investigations will be necessary to rule out the presence of potential virulence factors before considering its development as a safe candidate for functional food or therapeutic use.

## 5 Conclusion

This study discovered that the extract of *Kosakonia* sp. ZO-Rh4 has anti-diabetic properties both *in vitro* and *in vivo*, as well as the capacity to prevent diabetes complications—the safety of *Kosakonia* sp. ZO-Rh4 extract was proven through acute and sub-chronic toxicity testing in mice, which exhibited no adverse effects. Overall, our findings suggest that *Kosakonia* sp. ZO-Rh4 is relatively safe and has natural benefits for people.

## CRediT authorship contribution statement

**Trang Thi Xuan Dai:** Writing – review & editing, Writing – original draft, Visualization, Supervision, Resources, Funding acquisition, Data curation, Conceptualization. **Tran Chi Linh:** Methodology, Investigation. **Ta Lam Tai:** Methodology, Investigation.

## Ethical statement

The manuscript contains experiments using animals. The manuscript does not contain human studies. Approval for the study was granted by the Animal Ethics Committee of Can Tho University (CTU-AEC) under permission code CTU-AEC240001.

## Declaration of generative AI and AI-assisted technologies in the writing process

None.

## Funding information

This study was funded by the 10.13039/501100005645Ministry of Education and Training of Vietnam (Grant numbers B2023-TCT-02).

## Declaration of competing interest

The authors declare that they have no known competing financial interests or personal relationships that could have appeared to influence the work reported in this paper.

## Data Availability

Data will be made available on request.
